# P322: Study on clinical correlations, biological, and reducing the morbidity of malaria in Abidjan suburb of Abobo

**DOI:** 10.1186/2047-2994-2-S1-P322

**Published:** 2013-06-20

**Authors:** E Borges Pedro

**Affiliations:** 1 Association of Private Clinics in Côte d'Ivoire (ACPCI) - BERD SA, Abidjan, Côte d'Ivoire

## Objectives

Malaria is a serious public health problem. Should remember that only the female Anopheles is hematophagous. So there is a circulation of Plasmodium between men, between animals, between humans and animals and vice versa. The fact of non-human reservoir of Plasmodium is of major importance in any area where an eradication project is envisaged.

## Methods

Included among the priorities of the Constituent Assembly of the WHO in 1948, malaria was however widespread and increased in absolute and relative numbers in populations, particularly in Africa

## Results

The degree of immunity of hosts and reservoirs must necessarily be taken into account in the design and evaluation of malaria eradication programs. The clinical expand, with major changes, depending on the level of involvement of different pathophysiological mechanisms in action and the type of electrolyte disturbances. Brain suffering is due to general hypoxia and regional anoxia driven by a cytokine. Plasma levels of tumor necrosis factor are increased even more pernicious is that serious. It is anchored on these basis of evidence that the Program Perbor proposed the PGA-Global Programme of antimalarial systematic intervention by circles extensive progressive from the population-dense centers.

## Conclusion

The implementation of the Global antimalarial Programme, supported by education, training, monitoring and control of vectors of human and nonhuman populations of Plasmodium carriers can bring the truly successful fight against malaria.

## Disclosure of interest

None declared

